# Wind-triggered Antarctic sea-ice decline preconditioned by thinning Winter Water

**DOI:** 10.1038/s41558-026-02601-4

**Published:** 2026-03-18

**Authors:** Theo Spira, Marcel du Plessis, F. Alexander Haumann, Isabelle Giddy, Aditya Narayanan, Alessandro Silvano, Sebastiaan Swart

**Affiliations:** 1 Department of Marine Sciences, University of Gothenburg, Gothenburg, Sweden; 2 Alfred Wegener Institute, Helmholtz Centre for Polar and Marine Research, Bremerhaven, Germany; 3 Ludwig-Maximilians-Universität München, Munich, Germany; 4 Ocean and Earth Science, National Oceanography Centre, University of Southampton, Southampton, UK; 5 Department of Oceanography, University of Cape Town, Rondebosch, South Africa

**Keywords:** Physical oceanography, Cryospheric science, Climate-change impacts

## Abstract

Between 2015 and 2017, Antarctic sea ice underwent a drastic shift from a record high to a record low in sea ice area. While intensified atmospheric circulation and warmer upper-ocean temperatures in 2016 have been cited as possible causes for this sea ice regime shift, the contemporaneous subsurface ocean state remains poorly characterized. Here, using ~110,000 hydrographic profiles from the seasonally ice-covered Southern Ocean and atmospheric reanalysis, we show that a change in ocean–sea ice state was preconditioned by a thinning of Antarctic Winter Water between 2005 and 2015, while the reservoir of warmer deep water moved closer to the surface and sea ice. Then, in 2015, anomalously strong winds enhanced mixing across the thin Winter Water layer, entraining warm and salty subsurface waters, which broke down upper-ocean stratification. This combination of decadal-scale oceanic preconditions and strong wind-driven mixing in 2015 drove the sea ice loss that marked the regime shift.

## Abstract (web summary)

Antarctic sea ice declined sharply between 2015 and 2017, and this study uses ocean observations and atmospheric data to determine contributing factors. The authors show that thinning of Winter Water in the previous decade, followed by strong winds, brought warm deep water into contact with sea ice.

## Main

**Fig. 1:**
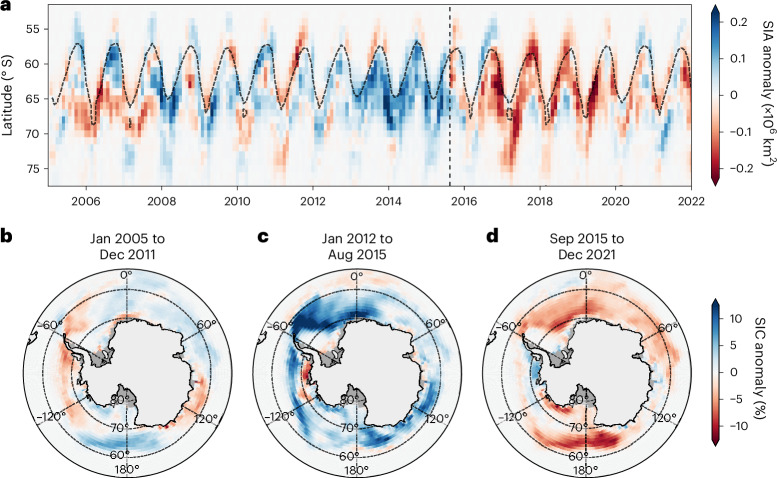
Southern Ocean sea ice anomalies from 2005 to 2022. **a**, Southern Ocean zonal mean of monthly SIA anomalies relative to the period 2005–2022. The thin dashed curve shows the mean 15% SIC and the vertical dashed black line denotes the transition from high SIA to low SIA (August 2015). **b**–**d**, The mean SIC anomaly from January 2005 to December 2011 (**b**), January 2012 to August 2015 (**c**) and September 2015 to December 2021 (**d**), respectively. Basemaps in **b**–**d** generated with Cartopy using data from Natural Earth and Openstreetmap under an Open Data Commons Open Database License (https://opendatacommons.org/licenses/odbl).

**Extended Data Fig. 1:**
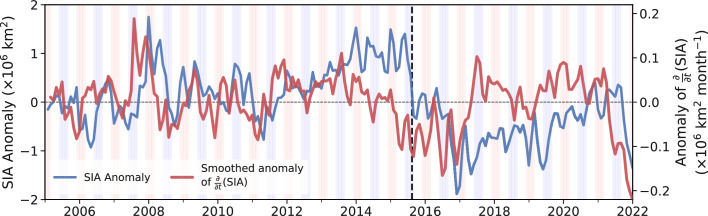
Sea ice area changes. Cumulative sea ice area anomaly (blue), the anomaly of the rate of change in time of cumulative sea ice area (red), relating to the right-hand y-axis, which has been smoothed using a 12-month rolling mean. The background blue and red stripes represent austral winter (JJA) and summer (DJF), respectively. The vertical dashed black line denotes the start of the transition from high SIA to low SIA (August 2015).

The Antarctic sea ice area (SIA) has undergone dramatic changes in recent decades^1–4^. From 2008 to 2015, the SIA was at a record high due to strong winds advecting ice equatorwards^5,6^ (Fig. 1a–c), reinforced by elevated ice shelf melt^7,8^ and sea ice–ocean feedbacks^9,10^. Then, in August 2015, an anomalously high SIA and early maximum in sea ice thickness^11^ were followed by an unexpected and abrupt decline in SIA (Fig. 1 and Extended Data Fig. 1), which continued to decrease until reaching a record minimum in summer 2016–2017^12^. This decline marked the onset of persistently low SIA from 2016 to the present, with recurring record lows^4^ (Fig. 1) potentially indicative of a new sea ice state^1–3,13,14^. These SIA changes cannot be explained by large-scale climate modes and numerical models struggle to reproduce the timing and magnitude of the observed changes in SIA^15–18^. Subsurface ocean heat has been implicated in the recent sea ice changes^3^; however, the causes and impact on SIA remain poorly understood.

Between 1981 and 2011, long-term hydrographic observations show warming (by 0.6 °C) and salinification (by 0.06) of Circumpolar Deep Water (CDW; ~200–600 m) while the ocean surface cooled (by 0.2 °C) and freshened (by 0.08)^9^. This accumulated heat in the ocean interior was likely entrained into the ocean surface layer, contributing to the reduction in SIA between 2015 and 2017^3,14,19^. Indeed, a coupled ocean–atmosphere model suggests that the ocean subsurface played a role in sustaining sea ice lows since 2016^14^. However, the causes of this subsurface heat release and their role in the abrupt, sustained sea ice loss have yet to be understood. In particular, the influence of upper-ocean salinity, which governs density^20^, stratification^21^ and water mass structure^22^ in the polar Southern Ocean, is poorly constrained from observations^14,23,24^.

**Fig. 2:**
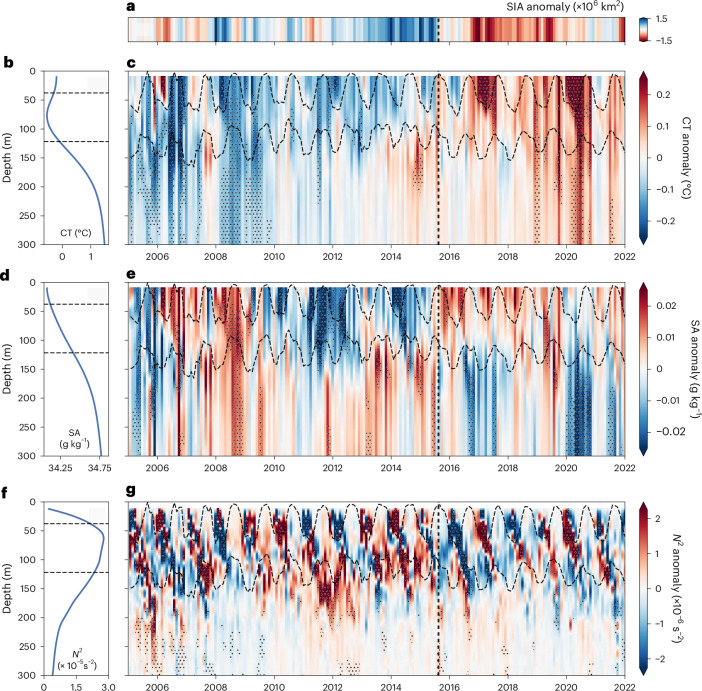
Vertical hydrographic anomalies. **a**, The monthly Southern Ocean SIA anomaly. **b**–**g**, The spatially weighted mean (**b**, **d** and **f**) and monthly anomaly profiles (**c**, **e** and **g**) of conservative temperature (CT; **b** and **c**), absolute salinity (SA; **d** and **e**) and Brunt-Väisälä frequencyBrunt-Väisälä frequency (stratification) (N^2^; **f** and **g**) in the seasonally ice-covered Southern Ocean. The vertical dashed black lines in **a**, **c**, **e** and **g** denote the start of the transition from high SIA to low SIA (August 2015). The stippling in **c**, **e** and **g** indicates regions where the anomaly is more than one standard deviation for each depth level. The dashed black horizontal lines in **b**–**g** denote the upper and lower WW boundaries.

Here, we focus on changes in Antarctic Winter Water (WW) and CDW, and their impacts on sea ice cover in the Southern Ocean. CDW is a relatively warm (~1–2 °C) and salty water mass that upwells south of the Polar Front^25,26^. WW, formed in the deep, cold wintertime mixed layer (ML) south of the Polar Front^27,28^, mixes with CDW to maintain upper-ocean stability via ocean–ice salinity and freshwater feedbacks^29^. In summer, a warm and fresh surface layer sits on top of WW^27,28,30–32^. Elevated stratification along the upper and lower boundaries of the WW acts as a barrier between the ML and CDW^31,33^ (Fig. 2f). Therefore, changes in the vertical structure and horizontal extent of WW regulate the upward access of warm CDW to the surface, directly impacting SIA.

We show that between 2005 and 2015, WW gradually thinned, while warm and salty CDW progressively shoaled towards the ocean surface. Subsequently, in winter 2015, anomalously strong winds elevated ocean mixing, entraining CDW into the ML and supplying heat that melted sea ice. This event inhibited further sea ice growth through a sustained reduction in upper-ocean stratification, ultimately changing the coupling between sea ice and CDW.

## Synchronous reversal of sea ice and upper-ocean anomalies

**Fig. 3:**
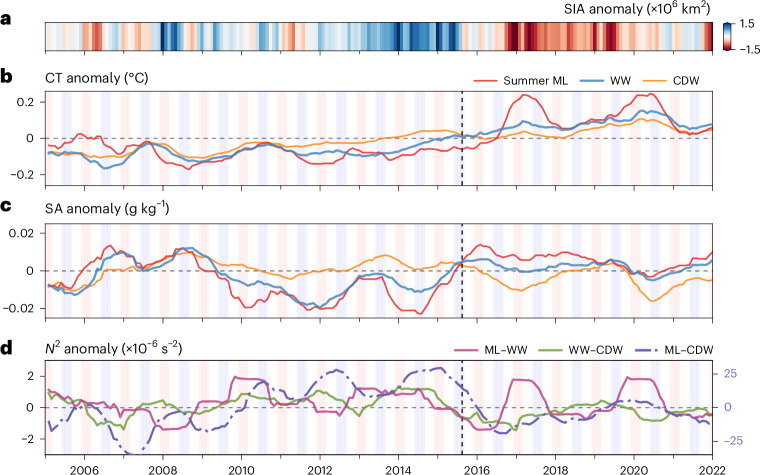
Layer-averaged hydrographic anomalies of water mass properties. **a**, The monthly Southern Ocean SIA anomaly. **b**–**d**, The spatially weighted 12-month rolling mean of CT (**b**), SA (**c**) and Brunt-Väisälä frequency (stratification) (**d**) anomalies. The red, blue and yellow lines in **b** and **c** show the vertically averaged anomalies for the summertime ML, WW and CDW, respectively. In **d**, the purple and green full lines show the stratification across the top and bottom of the WW boundary, that is, the ML–WW boundary and WW–CDW boundary, respectively. CDW is defined as from the WW lower boundary to 300 m. The dot-dashed indigo line shows the stratification difference between the mean summertime ML and mean CDW, and is measured using the right-hand *y* axis. The background blue and red stripes represent austral winter (JJA) and summer (DJF), respectively. The vertical dashed black line denotes the start of the transition from high SIA to low SIA (August 2015).

**Extended Data Fig. 2:**
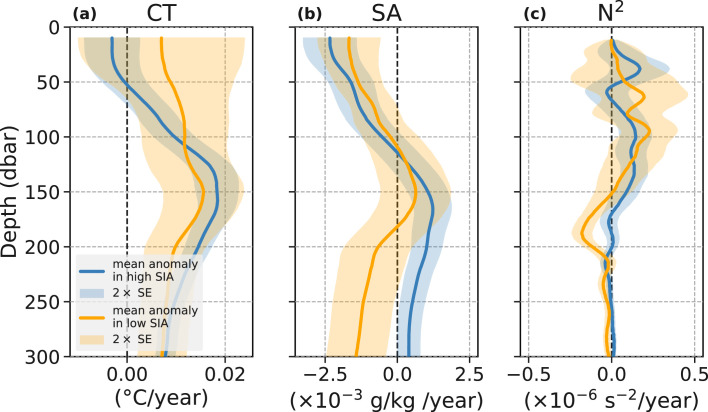
Trends of Southern Ocean properties for each depth level. (**a**–**c**) Yearly trend profiles of temperature, salinity and stratification, respectively. Blue and orange lines represent the periods of high sea ice area between Jan 2005 and August 2015 in blue and low sea ice area between September 2015 and December 2021 in orange. Shaded regions are two times the standard error.

Changes in SIA coincided with basin-scale shifts in the upper 300 m hydrography of the seasonally ice-covered Southern Ocean (Figs. 2 and 3 and Extended Data Fig. 2). During a period of increasing SIA (2008–2015), ML and WW temperatures typically remained cooler (−0.04 °C; Figs. 2c and 3b and Extended Data Fig. 2a) and fresher (~0.02 g kg^−1^; Figs. 2e and 3c and Extended Data Fig. 2b) than average. Meanwhile, CDW (from the WW bottom boundary to 300 m) warmed between 2009 and 2015 by ~0.15 °C (Figs. 2c and 3b and Extended Data Fig. 2a) and increased in salinity to ~0.005 g kg^−1^ above the mean in 2013–2015 (Fig. 3c and Extended Data Fig. 2b), which strengthened density gradients between the ocean layers (Figs. 2g and 3d). This pattern abruptly reversed in winter 2015. The ML and WW increased in salinity, whereas CDW freshened (Figs. 2e and 3c). Consequently, stratification weakened across both the ML–WW interface and the WW–CDW interface (*z*_WW−CDW_; Figs. 2g and 3d). Concurrently, SIA rapidly declined (Fig. 2a).

**Extended Data Fig. 3:**
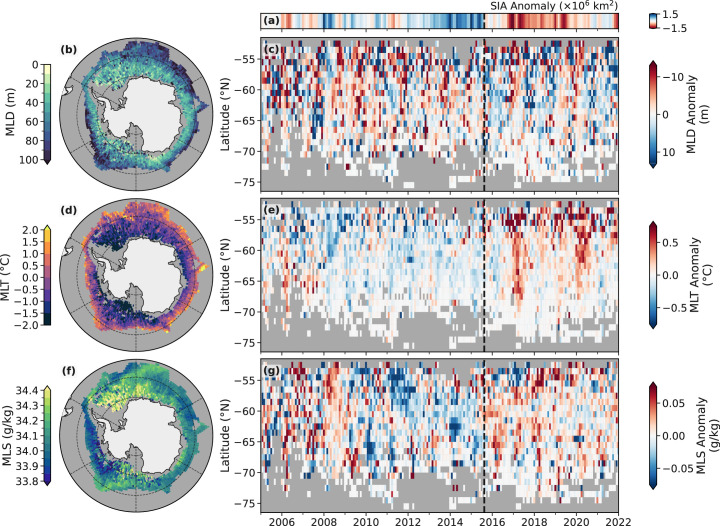
Anomalies of mixed layer properties. (**a**) Monthly Southern Ocean sea ice area anomaly. (**b**,**d**,**f**) Mean and (**c**,**e**,**g**) monthly anomaly mixed layer depth, mixed layer temperature, mixed layer salinity, respectively. The vertical dashed black line in the second column denotes the start of the transition from high SIA to low SIA (August 2015). Basemaps in **b**, **d** and **f** generated with Cartopy using data from Natural Earth and Openstreetmap under an Open Data Commons Open Database License (https://opendatacommons.org/licenses/odbl).

From the first SIA minimum in summer 2016–2017 onward, the entire upper 300 m warmed by ~0.1 °C (Figs. 2c and 3b). The changes in salinity persisted across large meridional extents of the Southern Ocean (Extended Data Fig. 3g), consistent with the weakened upper-ocean stratification^24^. Notably, there were two periods of strong stratification at the ML base in 2017 and 2020, which were summertime events associated with anomalously warm MLs across the Southern Ocean (>0.5 °C (ref. ^34^)) (Figs. 2c and 3b,d and Extended Data Fig. 3e). The synchronous changes in the vertical hydrographic structure of the upper Southern Ocean mark a shift that coincided with, and probably facilitated, the transition from high SIA to low SIA after 2015.

## Ocean preconditioning through WW thinning

**Fig. 4:**
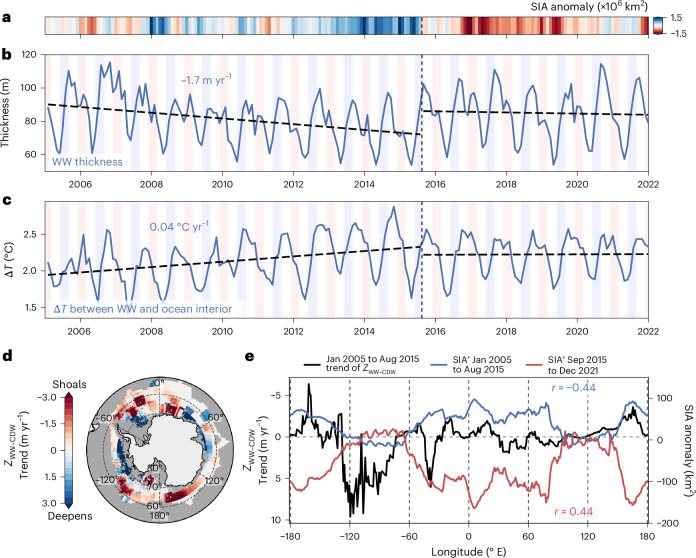
Antarctic WW changes. **a**, The monthly Southern Ocean SIA anomaly. **b**, The mean WW thickness. **c**, The mean temperature gradient across the WW–CDW interface. In **b** and **c**, the background blue and red stripes represent austral winter (JJA) and summer (DJF), respectively, and the horizontal dashed black lines are linear regressions computed before and after August 2015, with the trends from January 2005 to August 2015 annotated. The vertical dashed black line denotes the start of the transition from high SIA to low SIA (August 2015). **d**, Linear trend of the WW–CDW interface depth (*z*_WW–__CDW_) between January 2005 and August 2015, smoothed with a centred rolling mean (10° in longitude, 5°C in latitude). **e**, The meridional mean of the smoothed linear trend of the WW–CDW interface depth (black) and of the time-averaged SIA anomaly for the periods January 2005 to August 2015 (blue) and September 2015 to December 2021 (red). We correlate the two periods of mean SIA anomalies with the January 2005 to August 2015 trend of the WW–CDW interface depth, and present the Pearson’s *r* correlation for each period in their respective colours. The *P* value for each correlation, with adjusted degrees of freedom based on the 10° rolling mean, is ~10^−3^. Basemap in **d** generated with Cartopy using data from Natural Earth and Openstreetmap under an Open Data Commons Open Database License (https://opendatacommons.org/licenses/odbl).

**Extended Data Fig. 4:**
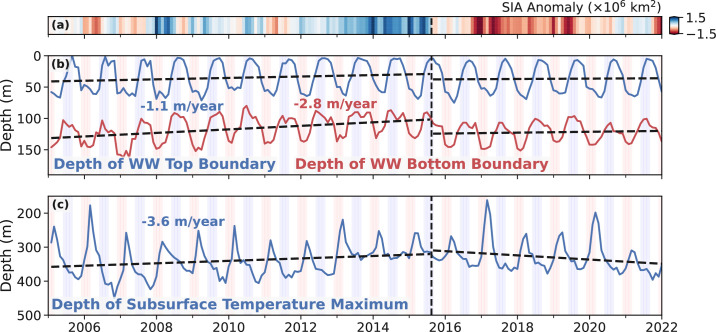
Winter Water boundaries and subsurface heat reservoir. (**a**) Monthly Southern Ocean sea ice area anomaly. (**b**) Depth of the Winter Water top and bottom boundaries in blue and red, respectively. (**c**) Depth of the subsurface temperature maximum. In (**b**) and (**c**), the background blue and red stripes represent austral winter (JJA) and summer (DJF), respectively, and means are spatially weighted. The horizontal black dashed lines are linear regressions computed before and after August 2015, with the trends annotated. The vertical dashed black line denotes the start of the transition from high SIA to low SIA (August 2015).

Between 2005 and 2015, WW thinned at a rate of 1.7 m yr^−1^ (Fig. 4b) and thus reduced in thickness by 20%. This thinning was largely attributed to the shoaling of the WW bottom boundary (2.8 m yr^−1^), while the WW top boundary shoaled more slowly (1.1 m yr^−1^; Extended Data Fig. 4b). The thinning of WW was likely driven by the subsurface heat reservoir rising closer to the surface, with the subsurface temperature maximum ascending at 3.6 m yr^−1^ across the same period (Extended Data Fig. 4c).

The thinning of WW from below is driven by various mixing processes that act to erode WW across a typical annual cycle^31^. Given that WW is a cold layer that stably overlays warm CDW in the ocean interior (Fig. 2b), changes in the vertical temperature gradient between WW and CDW alter down-gradient heat fluxes^31,35,36^. Between 2005 and 2015, WW thinned from below (that is, shoaling of the WW–CDW interface, *z*_WW−CDW_; Extended Data Fig. 4b), which coincided with an increase in the vertical temperature gradient across this interface (Fig. 4c). Between 2010 and 2015, the maximum vertical temperature gradient across *z*_WW−CDW_ was ~2.14 °C in spring 2013. Furthermore, the minimum temperature gradient over the annual cycle increased consistently between 2005 and 2015, with a minimum of ~1.41 °C in 2007 to ~1.96 °C in 2013. The increased vertical temperature gradient is indicative of the continued accumulation of heat in the subsurface^9^ and shoaling of warm CDW^37^, as indicated by the subsurface temperature maximum (Extended Data Fig. 4c). Following the reduction of SIA in 2015, the positive trend in the vertical temperature gradient disappeared and the amplitude of its seasonal cycle reduced in magnitude (Fig. 4c), which may be connected to the subsequent shoaling of the subsurface temperature maximum (Extended Data Fig. 4c).

The linear trend of *z*_WW−CDW_ from January 2005 to August 2015 varies in sign and magnitude across the Southern Ocean, with large regions showing persistent shoaling exceeding 3 m yr^−1^ (Fig. 4b and red regions in Fig. 4d). In particular, *z*_WW−CDW_ shoaled across much of the Ross Sea (~−180° to −120° E), while it predominantly deepened in the Amundsen and Bellingshausen seas (−120° to −70° E) (Fig. 4d,e). In the Weddell Sea, trends varied meridionally, with deepening in the southern Weddell Sea and dominant shoaling in the northern parts, consistent with previous findings of regional CDW variability^37,38^. A similar deepening trend was observed near the eastern Antarctic continental shelf (~30° to 90° E). Importantly, the meridional mean of the *z*_WW−CDW_ 2005–2015 trend negatively correlates with mean SIA anomalies from January 2005 to August 2015 (*r* = −0.44, *P* ≪ 0.01), with regions of *z*_WW−CDW_ shoaling aligning with sea ice expansion (Fig. 4e). Then, the same trend positively correlates with SIA anomalies from September 2015 to December 2021 (*r* = 0.44, *P* ≪ 0.01), suggesting a fundamental change in regional ocean–ice coupling. In this later period, the initial long-term thinning of WW before 2015 appears to enhance vertical ocean–ice connectivity after 2015, allowing increased heat exchange between CDW and the surface, contributing to sustained sea ice loss. This sign reversal indicates that the WW–CDW interface no longer acted as a stratification barrier and, instead, facilitated upper-ocean warming. The correlation sign reversal between the *z*_WW−CDW_ 2005–2015 trend and SIA anomalies of the two periods further reinforces the interpretation of a shift in ocean–ice interactions. However, the mechanism that triggered the observed changes in upper-ocean properties and connectivity with sea ice remains unclear.

## Wind-driven trigger in 2015

**Extended Data Fig. 5:**
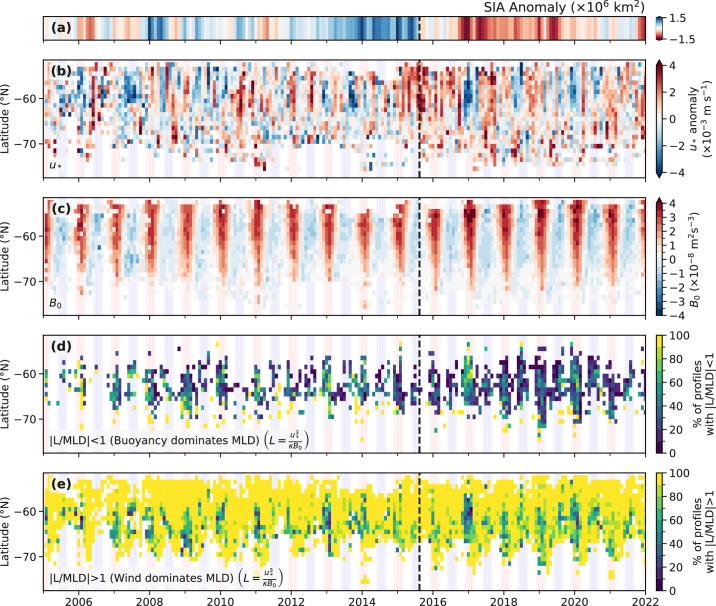
Monin–Obukhov length in the Southern Ocean seasonal ice zone. (**a**) Monthly Southern Ocean sea ice area anomaly. (**b**) Frictional velocity anomaly. (**c**) Net surface buoyancy flux (excluding ice-ocean fluxes). (**d**,**e**) Monin-Obukhov length to mixed layer depth (MLD) ratio, showing the percentage of profiles for each latitude band where buoyancy dominates mixed layer depth variations such that ∣L/MLD∣ < 1, and where wind dominates mixed layer depth variations such that ∣L/MLD∣ > 1, respectively. The vertical dashed black line denotes the start of the transition from high SIA to low SIA (August 2015).

To determine the driver of the sudden, simultaneous shift of upper-ocean properties and SIA decline, we examined the oceanic surface forcing induced by wind. Specifically, we consider the friction velocity at the ocean surface, which depends on wind stress and can be used as a proxy for diapycnal mixing in the ML (Methods). This relationship has been confirmed in the Southern Ocean by correlating observed and theoretical dissipation rates^31,39,40^. We computed the Monin–Obukhov length (Methods), which suggests that ML turbulence in the partially ice-covered Southern Ocean in 2015 is dominated by mechanical rather than buoyancy-driven mixing (Extended Data Fig. 5). We find that friction velocity (that is, wind forcing) dominated turbulent dissipation in the ML across most of the seasonally ice-covered Southern Ocean, indicating that entrainment at the base of the ML was driven by mixing associated with strong winds, and accounted for >90% of ML turbulence generated during winter 2015 (Extended Data Fig. 5e).

**Fig. 5:**
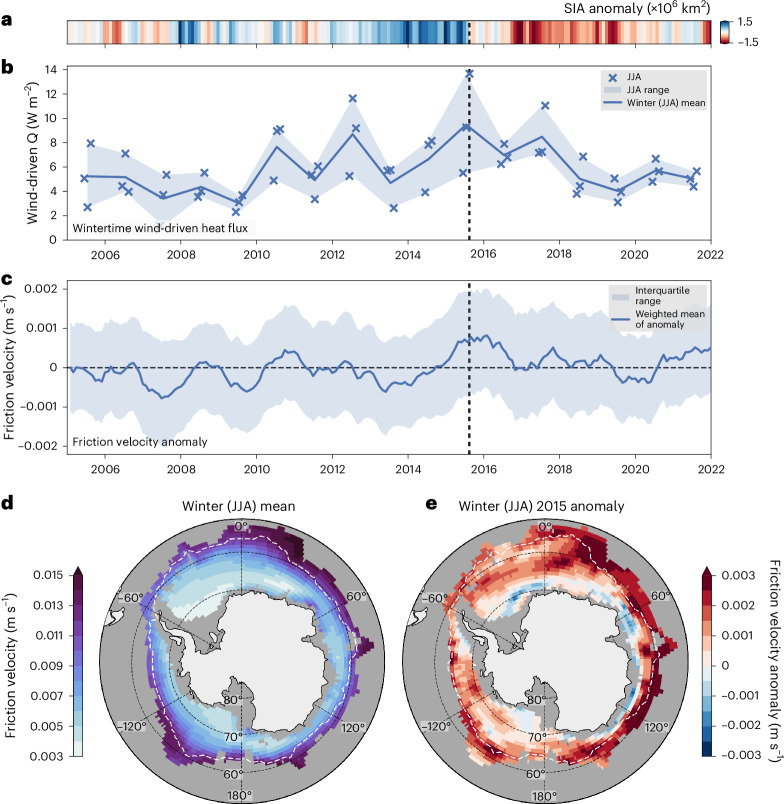
Enhanced wind-driven heat fluxes in winter 2015. **a**, The monthly Southern Ocean SIA anomaly. **b**, The spatially weighted mean of the wintertime wind-driven heat flux (*Q*). **c**, The spatially weighted mean of friction velocity monthly anomalies, with the interquartile range indicated by the shaded region. The line denotes the wintertime average, the markers denote the wintertime (JJA) monthly weighted means and the shaded region denotes the wintertime range. **d**,**e**, Climatological wintertime friction velocity (**d**) and winter 2015 friction velocity anomaly (**e**), respectively. The dashed white line denotes the 15% SIC line for the mean wintertime and winter 2015, respectively. The vertical dashed black lines in **a**–**c** denote the transition from high SIA to low SIA (August 2015). Basemaps in **d** and **e** generated with Cartopy using data from Natural Earth and Openstreetmap under an Open Data Commons Open Database License (https://opendatacommons.org/licenses/odbl).

Wind-driven subsurface heat fluxes also underwent significant changes. Wintertime averages were typically below 6 W m^−2^ between 2005 and 2010 (Fig. 5b), but gradually increased after that, reaching a maximum in August 2015 of ~14 W m^−2^ across the ice-covered Southern Ocean. Such a heat flux would be sufficient to warm the ML by ~0.13 °C for that month and melt an additional ~5 cm of sea ice per m^2^ on average or melt up to ~12 cm of ice per m^2^ without additional ML warming, suppressing the rate of sea ice formation and contributing to the anomalously low ice growth in late 2015 (Extended Data Fig. 1). This subsurface heat flux anomaly was accompanied by concurrent changes in the sign of the salinity anomaly between CDW and the upper ocean in August 2015 (Fig. 3c), indicating a vertical exchange of salinity between water masses.

**Extended Data Fig. 6:**
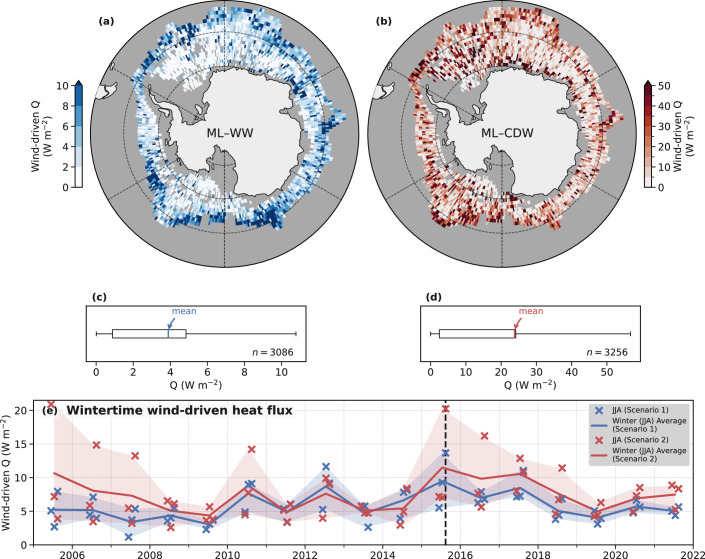
Wind-driven upward ocean heat flux into the mixed layer under different conditions. The annual mean wind-driven heat flux over the period January 2005-December 2021 under: (**a**,**c**) unadjusted oceanic conditions where the mixed layer (ML) typically resides above Winter Water (WW); and (**b**,**d**) adjusted oceanic conditions where Winter Water is artificially removed and the mixed layer resides directly above Circumpolar Deep Water (CDW). (**a**,**b**) show the time-mean wind-driven upward ocean heat flux (Q), and (**c**,**d**) are the respective spatially weighted boxplots of the time-mean heat fluxes. The boxplots are derived using spatially weighted statistics, where the box spans the interquartile range, the whiskers extend to the minimum and maximum of 1.5 × the interquartile range, and the coloured vertical bars indicate the mean with n=3086 and n=3256 (number of grid cells), respectively. (**e**) The spatially weighted mean austral winter (JJA) wind-driven heat flux for the unadjusted oceanic conditions (Scenario 1) in blue and the adjusted oceanic conditions where the mixed layer lies directly above Circumpolar Deep Water (Scenario 2) in red. The lines show the mean wintertime wind-driven heat flux, the crosses show the monthly average for wintertime, and the shaded region shows the range. A positive heat flux indicates heat fluxed into the mixed layer from below. Basemap in **a** generated with Cartopy using data from Natural Earth and Openstreetmap under an Open Data Commons Open Database License (https://opendatacommons.org/licenses/odbl).

Stratification plays an important role in modulating wind-driven heat fluxes. Thus, to contextualize the role of WW as a stratification barrier between warm CDW and the ML, we estimated the wind-driven heat fluxes under two scenarios: (1) an unadjusted water column where WW is below the ML (Fig. 5b) and (2) an adjusted water column without WW, where the ML resides directly above warm CDW (Extended Data Fig. 6). The mean wind-driven heat flux in scenario 2 was more than six times greater than scenario 1 (~24 W m^−2^ and ~4 W m^−2^, respectively; Extended Data Fig. 6c,d). This result highlights the role of WW in preventing upward heat transfer into the ML via wind-driven mixing.

Hence, sea-ice decline in 2015 was probably a result of atmospheric forcing, consistent with the simulations-based study of Zhang et al. ^14^. This forcing elevated upward oceanic heat fluxes into the ML and coincided with the observed breakdown of stratification in 2015—first across the ML–WW boundary, then across *z*_WW−CDW_ (Fig. 3d). A state of reduced stratification was subsequently sustained throughout the upper ocean from late 2015 onward (Figs. 2g and 3d).

## Discussion

**Fig. 6:**
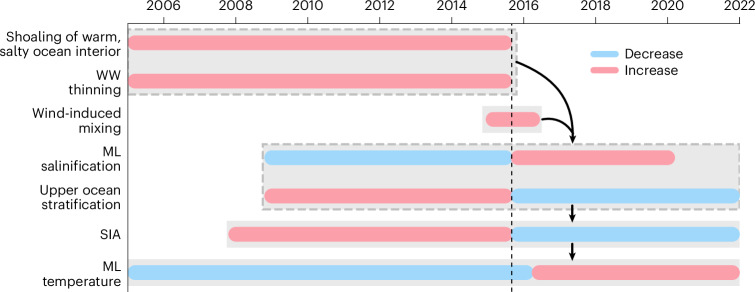
Timeline summarizing interacting ocean, wind and sea ice processes. The red and blue bars indicate an increase and decrease of the property, respectively. The grey boxes group processes that are linked and take place synchronously. The arrows indicate chronology between processes. The vertical dashed black line denotes the start of the transition from high SIA to low SIA (August 2015).

The low SIA anomalies have continued unabated from 2016 to the present day, with record-breaking sea ice minima in 2016, 2022 and 2023^1,3,4,41^. While several studies have indicated a potential regime shift to a state of low and more variable sea ice coverage^2–4^, the driving mechanisms and the timing of this change remained unclear, particularly in terms of the ocean’s role in this variability. Here, we provide observationally based insights into ocean property changes over a 17-year period (2005–2022), capturing the transition from satellite-observed record highs in SIA (2012–2015) to record lows (2016 to present day) (Fig. 1). Our analysis partially coincides with the period of an at least 30-year-long trend (1981–2011) of heat and salt accumulating in the ocean subsurface (where CDW resides), alongside freshening and cooling at the ocean surface^9,37^. From 2012 to 2015, these trends in the subsurface and surface intensified in concert with SIA increase, which elevated the upper-ocean stratification (Figs. 2 and 6).

We show that WW acts as a stratification barrier that considerably inhibits the upward transfer of heat from CDW into the ML (Extended Data Fig. 6). We also show that WW thinned from below due to the shoaling of warm, salty CDW (Figs. 4 and 6 and Extended Data Fig. 4) over a multiyear period (2005–2015). The shoaling of CDW was potentially driven by long-term variations in atmospheric circulation that increased ocean gyre cyclonicity and enhanced CDW upwelling^14,23,42,43^, consistent with observed multidecadal CDW shoaling trends across large regions of the polar Southern Ocean^37,38^. Elevated atmospheric and sea ice vorticity across the Southern Ocean from 1991 to 2020^44^ further suggest not only enhanced cyclonicity but also an increased rate of energy transfer via atmosphere–ice–ocean stresses. In addition, the strengthened vertical temperature gradient across the WW–CDW interface (Fig. 4c) likely enhanced diffusive mixing, further contributing to the thinning of WW^31,35^.

In 2015 and 2016, stronger-than-average winds persisted across the entire seasonally ice-covered Southern Ocean (Fig. 5c), in particular in austral winter (Fig. 5e). Combined with a thinner WW layer and shallower CDW layer, the wind-driven mixing resulted in a strong vertical flux that brought heat and salt into the ML (Fig. 5b) and weakened upper-ocean stability (Fig. 3g). Weakened stratification allowed for enhanced exchange of warm CDW into the ocean ML and elevated rates of sea ice melt (Fig. 6 and Extended Data Fig. 1).

The change in SIA between 2015 and 2017 coincided with a change in the upper-ocean hydrographic structure. Salinity in the WW and ML remained higher than average from late 2015 onward, concurrent with reduced salinity in CDW, decreasing the vertical density gradient. These salinity changes resulted in a sustained lower upper-ocean stratification^24^ (Figs. 2 and 3) and reduced the WW barrier effect. Consequently, there was increased connectivity between the upper ocean and ocean interior, enabling heat supply from below that continued to warm the upper ocean, suppressing sea ice growth and maintaining low SIA (Fig. 6). This altered upper ocean–sea ice connectivity is reflected by the reversal in correlation between the regional shoaling of the WW–CDW interface depth before 2015 and SIA anomalies before and after 2015 (Fig. 4e). The change in correlation sign indicates that sea ice coverage reduced in regions of prior CDW shoaling, suggesting a modified ocean–ice coupling.

**Extended Data Fig. 7:**
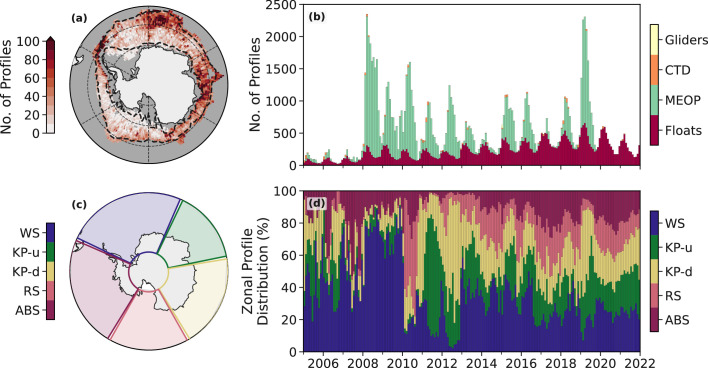
Hydrographic dataset in the seasonally ice-covered Southern Ocean. (**a**) Total number of profiles between 2005 and 2022. The black dashed line denotes the mean 15% sea ice concentration contour for February (month of minimum sea ice cover) and September (month of maximum sea ice cover). (**b**) The monthly total number of profiles, coloured by data source, shown as stacked bars. (**c**) The Southern Ocean separated into its regional sectors. (**d**) Regional distribution of all profiles per ocean sector as a percentage for all profiles. The labels in (**c**) and (**d**) denote the regions of the Weddell Sea (WS), upstream of Kerguelen Plateau (KP-u), downstream of Kerguelen Plateau (KP-d), the Ross Sea (RS) and the Amundsen and Bellingshausen Seas (ABS). Basemaps in **a** and **c** generated with Cartopy using data from Natural Earth and Openstreetmap under an Open Data Commons Open Database License (https://opendatacommons.org/licenses/odbl).

Understanding the recent drastic Antarctic sea ice loss is critical for various aspects of the Earth’s climate system, including its impact on atmosphere–ocean heat and carbon exchange^39,41,45–48^, the Earth’s albedo effect^49^, ocean circulation^22,50,51^ and the Antarctic marine ecosystem^52–54^. Using hydrographic ocean observations, we have identified and described an important process that may not be resolved in climate models, highlighting the need for sustained, long-term observations in the Southern Ocean and the crucial role of existing observing systems—such as float programmes and the Marine Mammals Exploring the Oceans Pole to Pole programme (Extended Data Fig. 7). We provide observational evidence that the ocean preconditioned sustained sea ice loss, which was ultimately triggered by strong winds in 2015 and culminated in a change in the ocean–ice state where WW has a reduced barrier effect, with greater connectivity between sea ice and the warm ocean interior. Nonetheless, further work is urgently needed to assess whether the Southern Ocean and Antarctic sea ice has, indeed, undergone a long-term regime shift.

## Methods

### Hydrographic observations and WW identification

Using 589,312 hydrographic profiles from around the circumpolar Southern Ocean (40° S and poleward) from 2005 to 2022^55^, we investigated changes to the upper ocean (top 300 m). We computed the ML depth (MLD) following a density difference criterion (*ρ*(*z*_MLD_) − *ρ*(10) = 0.03 kg m^−3^) (ref. ^56^).

We identified WW following Spira et al. ^28^, who characterize WW into two classifications: ML–WW (constrained to the ML) and subsurface–WW (constrained to below the ML if there is a temperature inversion below the ML, for example; Fig. 3b). The boundaries of subsurface WW are defined as the maximum temperature gradients above and below the temperature minimum. If the temperature at a boundary is >2 °C, then the boundary is taken as the 2 °C isotherm depth, which is an important constraint to avoid conflation of WW with the warmer subsurface ocean interior. This criterion is typically applied when the temperature gradient begins to erode in autumn or late in the annual cycle and is thereby important for the thickness and boundary depths^28^. WW has a maximum temperature of 2 °C to limit its northern extent to the polar front, which denotes the northern boundary where WW can no longer form—without this criterion, the WW identification algorithm aliases sub-Antarctic mode water as WW^28^. For simplicity, in this study, we do not distinguish between the two different WW types but use their combined occurrence. We label the lower boundary as the interface between WW and CDW (*z*_WW–CDW_). We also identify the subsurface temperature maximum as the maximum temperature below the ML, which is representative of the subsurface heat reservoir associated with CDW^57^.

Hydrographic properties are computed per profile, then gridded to a 1° by 1° latitude–longitude median monthly time series. As we are interested in the interaction between the ocean and the sea ice, we select all profiles from the seasonally ice-covered Southern Ocean (Extended Data Fig. 7a). The WW core temperature is the minimum temperature in the WW profile^28^, which, at 0 °C, is representative of the region where sea ice can form (Extended Data Fig. 7a) and provides an estimate of the northern bound of the seasonally ice-covered Southern Ocean. Thus, we define the seasonally ice-covered region as poleward of the mean 0 °C WW core temperature isotherm south of the polar front. We only investigate regions where the bathymetry is deeper than the 2,000 m isobath, removing shelf regions that exhibit different hydrographic dynamics. Consequently, we investigate the hydrographic changes in the remaining 109,689 profiles, of which 101,230 (92%) contain WW and 8,459 profiles do not contain WW (unclassified). Hydrographic profiles are not homogeneously distributed in time and space (Extended Data Fig. 7b,c). This distribution is partially a result of the considerable differences in area size per ocean sector (Extended Data Fig. 7c). Nonetheless, observations cover all ocean sectors and are relatively consistent in their bulk regional coverage (Extended Data Fig. 7d), providing reasonable data for the quantification of circumpolar changes in time.

### Ocean anomalies and trends

Following gridding of hydrographic profiles, we compute a monthly anomaly ${X}^{{\prime} }=X-\overline{X}$, where $\overline{X}$ is the climatological monthly mean for each grid cell and depth level (10–300 m). To account for varying grid cell sizes across latitudes, we compute spatially weighted means (${\bar{X}}_{w}=\frac{{{\rm{\sum }}}_{i}{w}_{i}{X}_{i}}{{{\rm{\sum }}}_{i}{w}_{i}}$) and spatially weighted standard deviations (${\sigma }_{w}=\sqrt{\frac{{{\rm{\sum }}}_{i}{w}_{i}{({X}_{i}-{\bar{X}}_{w})}^{2}}{{{\rm{\sum }}}_{i}{w}_{i}}}$) for some variable *X*_*i*_ in grid cell *i* and grid cell area *w*_*i*_. We find that trends during the period of high SIA (January 2005 to August 2015) are robust, with temperature trends of −0.004 °C yr^−1^ in the summertime ML, 0.004 °C yr^−1^ in the WW layer and warming of CDW by 0.012 °C yr^−1^ (Figs. 2c and 3b and Extended Data Fig. 2a). Similarly, across the same period, we find that the ocean surface and WW freshened, while CDW salinified with trends of −2.2, −0.98 and 0.83× 10^−3^ g kg^−1^ yr^−1^, respectively (Figs. 2e and 3c and Extended Data Fig. 2b), agreeing with previous findings^9^. These trends are robust, with *P* values <0.03 for each of the vertical ocean sections (as in Fig. 4) with the exception of CDW, which had a *P* value of 0.23. When SIA was low (August 2015 to December 2021), the entire upper-ocean vertical temperature profile exhibited a warming trend (~0.01 °C yr^−1^; Extended Data Fig. 2a), and the entire salinity profile exhibited a freshening trend (−0.81 × 10^−3^ g kg^−1^ yr^−1^; Extended Data Fig. 2b). Salinity *P* values for each of the vertical ocean sections are largely <0.04, with the exception of WW, which has a value of 0.16. Summertime ML and CDW temperature *P* values are 0.39 and 0.48, respectively, while WW has a *P* value of <0.003.

### Computation of wind-driven heat fluxes

To understand the possible effect of wind-driven mixing on the water column, we here estimate turbulent dissipation rates. There is a strong correlation between theoretical and observed turbulent dissipation rates in the Southern Ocean boundary layer in summertime^31,39,40^, which we apply to all seasons. Given the spatial and temporal sparsity of dissipation observations, it is not clear whether this relationship holds under different oceanic and climatic conditions, so the results must be interpreted with caution. Thus, turbulent dissipation can be approximated from the kinematic wind stress following the law of the wall^58^ such that dissipation scales with friction velocity and distance from the boundary: $\varepsilon =\frac{{u}_{* }^{3}}{kz}$ where *k* = 0.41 is von Kármán’s constant, *z* is depth (positively increases with depth) and is taken here as the the ML depth and friction velocity, ${u}_{* }=\sqrt{\frac{\tau }{{\rho }_{0}}}$. From dissipation, we can estimate the diffusive rate of exchange of properties across density gradients (diapycnal diffusivity) via $\kappa =\Gamma \frac{\varepsilon }{{N}^{2}}$ where *N*^2^ is the stratification across the base of the ML. We use diapycnal diffusivity to approximate the wind-driven turbulent heat flux $Q=\frac{{\rho }_{0}\,{C}_{{\rm{p}}}}{z}\,\kappa \,\Delta T$ where sea water density *ρ*_0_ = 1,035 kg m^−^^3^ and specific heat capacity *C*_p_ = 3,850 J kg^−1^ K^−1^ (refs. ^28,39^). We can estimate the loss of sea ice thickness from the wind-driven upward oceanic flux using the latent heat of sea ice using $\Delta {h}_{\mathrm{SI}}=\frac{Q\,\Delta t}{{\rho }_{{\rm{i}}}\,{L}_{{\rm{f}}}},$ where Δ*h*_SI_ is the change in sea ice thickness, Δ*t* is the change in time (s), *ρ*_i_ = 917 kg m^−3^ is the density of sea ice and *L*_f_ = 334, 000 J kg^−1^ is the latent heat of fusion for sea ice. In ice-covered regions, we modify momentum transfer by following a combination of the ice-ocean stress ‘rule of thumb’ that imposes a one-third magnitude of atmosphere–ocean stress (*τ*_ao_) in regions of ice cover^59^ in combination with a calculation of momentum transfer from ocean to atmosphere accounting for sea ice concentration (SIC) and sea ice velocity^60^. We combine these two approaches such that $\tau =(1-\frac{2}{3}A){\tau }_{\mathrm{ao}}$, where *τ*_ao_ is obtained from turbulent surface stress product output^61^ and *A* is the SIC.

This dissipation–wind relationship holds under the assumption that wind is the main driver of turbulence in the ML. We support this assumption through the computation of Monin–Obukhov length, $L=\frac{-{u}_{* }^{3}}{kB}$, where *k* is Von Kármán’s constant and *B* is the net surface buoyancy forcing. The Monin–Obukhov length approximates the depth of turbulent mixing from surface forcing^62,63^. When *z* is equal to the ML depth, the Monin–Obukhov length provides context to the processes dominating the turbulent kinetic energy content in the surface ocean^31^ (Extended Data Fig. 5).

### Other datasets

To define the polar front of the Antarctic Circumpolar Front, we use a height of −0.58 m of the absolute dynamic topography^64^ from monthly AVISO altimetry data. We use bathymetric data from the International Bathymetric Chart of the Southern Ocean^65^ and base maps from Cartopy^66^. The components of the net surface heat flux (comprising net shortwave radiation, *Q*_SW_, net longwave radiation, *Q*_LW_, latent heat flux, *Q*_lat_, and sensible heat flux, *Q*_sen_), precipitation and evaporation, and the eastward and northward turbulent wind stresses for the computation of friction velocity are obtained at monthly and 0.25° resolution from ERA5 reanalysis of the European Centre for Medium-Range Weather Forecasts^61^ and are interpolated onto a mean 1° by 1° resolution for co-location with hydrographic gridding. Using monthly, 25 km × 25 km SIC data^67^, we computed SIA by multiplying the SIC with the grid-cell area. These data were interpolated to a monthly 1° by 1° grid and used to compute the total sum over the Southern Ocean.

## Online content

Any methods, additional references, Nature Portfolio reporting summaries, source data, extended data, supplementary information, acknowledgements, peer review information; details of author contributions and competing interests; and statements of data and code availability are available at 10.1038/s41558-026-02601-4.

## Data Availability

We followed the methodology and code of Spira et al. for the identification and classification of Antarctic WW^28,68^. The hydrographic profile dataset is openly available via Zenodo at https://zenodo.org/records/10258138 (ref. ^55^).

## Code availability

Analysis and visualization code for this work is openly available via GitHub at https://github.com/theospira/WW-sea_ice (ref. ^69^).
